# Supplementary material for: Splice-Junction-Based Mapping of Alternative Isoforms in the Human Proteome
Source: Cell Rep. Author manuscript; Available in PMC 2020 Jan 15. (PMC6961840; doi:10.1016/j.celrep.2019.11.026)
Supplement: 2 [file NIHMS1546469-supplement-2.zip › DF1/PXD006675/LeftAtrium/LeftAtrium_14_PPP1R1A_ELQMMVEHHLGQQQQGEEPEGAAESTGTQESRPPGIPDTEVESR.pdf]

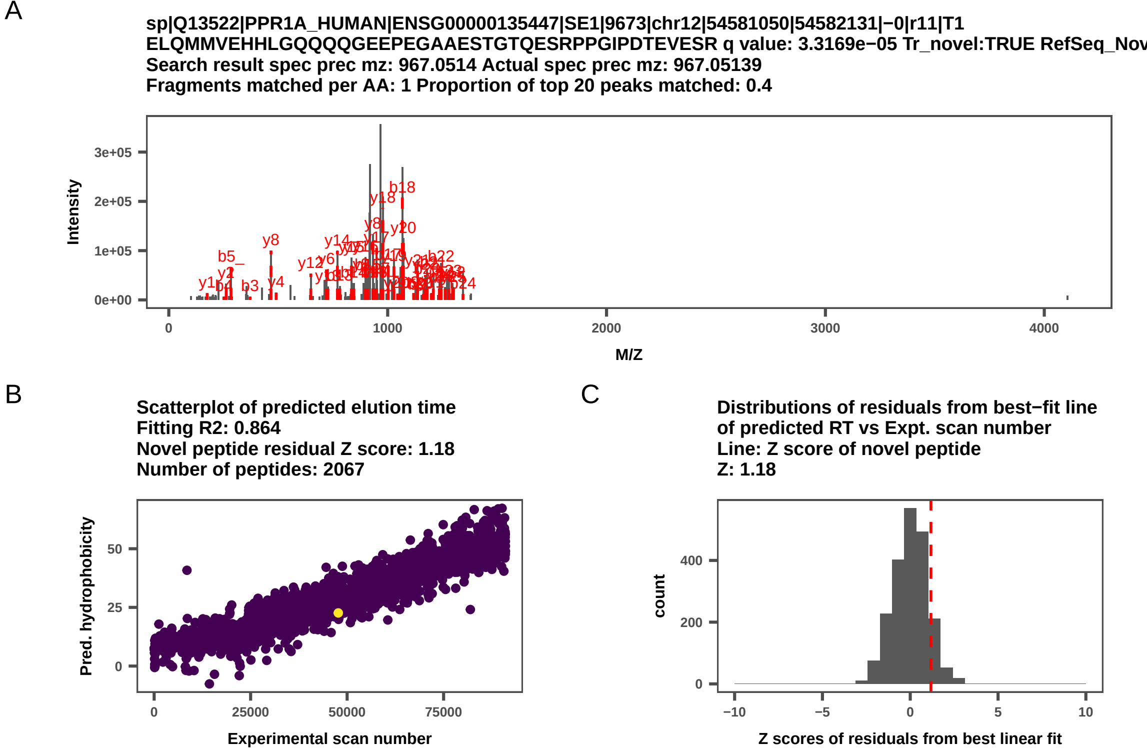
A
sp|Q13522|PPR1A_HUMAN|ENSG00000135447|SE1|9673|chr12|54581050|54582131|−0|r11|T1
ELQMMVEHHLGQQQQGEEPEGAAESTGTQESRPPGIPDTEVESR q value: 3.3169e−05 Tr_novel:TRUE RefSeq_Nov
Search result spec prec mz: 967.0514 Actual spec prec mz: 967.05139
Fragments matched per AA: 1 Proportion of top 20 peaks matched: 0.4
Intensity
3e+05
2e+05
1e+05
0e+00
b18
y18
y8
y20
y8
y14
y12
y6
b5_
y2
y1
b1
b3
y4
b22
b24
0
1000
2000
3000
4000
M/Z
B
Scatterplot of predicted elution time
Fitting R2: 0.864
Novel peptide residual Z score: 1.18
Number of peptides: 2067
Pred. hydrophobicity
50
25
0
0
25000
50000
75000
Experimental scan number
C
Distributions of residuals from best−fit line
of predicted RT vs Expt. scan number
Line: Z score of novel peptide
Z: 1.18
count
400
200
0
−10
−5
0
5
10
Z scores of residuals from best linear fit
